# Prevalence and correlates of anxiety and depression among family carers of cancer patients in a cancer care and treatment facility in Uganda: a cross-sectional study

**DOI:** 10.4314/ahs.v17i3.30

**Published:** 2017-09

**Authors:** Godfrey Katende, Lillian Nakimera

**Affiliations:** 1 Sultan Qaboos University, Nursing; Makerere University, Nursing; 2 Makerere University College of Health Sciences, Department of Nursing

**Keywords:** Anxiety, depression, cancer patients, Uganda

## Abstract

**Background:**

The process of caregiving may cause emotional distress in form of anxiety and depression among family carers of cancer patients. Little is known about the prevalence of anxiety and depression among family carers of cancer patients in Uganda.

**Objectives:**

To determine the prevalence of anxiety and depression and related factors associated with abnormal levels of anxiety and depression among family carers of cancer patients in a cancer care and treatment facility in Uganda.

**Methods:**

After obtaining ethical approval, we recruited family carers of cancer patients to this cross-sectional study. Data was collected with the use of the Hospital Anxiety and Depression Scale(HADS) standardized tool.

**Results:**

A total of 119 family carers were recruited from the Uganda Cancer Institute. The prevalence of anxiety and depression among family carers was high (45% V. 26 %); Abnormal levels of anxiety (ALA)(OR 0.27, 95% CI, p= 0.01) and depression (ALD)(OR 0.37, 95% CI, p=0.05) were significantly associated with being a relative carer.

**Conclusion:**

Anxiety and depression is prevalent among family carers of cancer patients. Being a relative carer predisposes you to increased risk of developing anxiety and depression. Incorporating evidence based psychological therapies into usual care and targeting family carers is imperative.

## Introduction

Family caregivers of cancer patients experience multiple stressors in the form of emotional distresses. The caring role of cancer patients can pose a significant burden to family carers^1^. Carers of cancer patients are likely to report feelings of being overwhelmed, mood changes, anxiety and depressive disorders^2^,^3^. Studies indicate that anxiety and depression not only do they affect cancer patients but also affect negatively their family carers^4^. Stressful circumstances, such as generalized anxiety disorder, panic attacks and post-traumatic stress disorder are likely to be present in family carers of cancer patients^5^. Similarly, depression among family carers may be present by manifesting as feelings of loneliness, isolation, fearfulness, and irritability^6^. Studies have indicated that 50% of the people with depression do not receive any treatment for their depression in low- and middle-income countries thus increasing the depressive burden in these countries^7^.

Cancer mortality rates are documented to be on the rise with one in five deaths occurring in Africa^8^,^9^. In 2007, cancer death rates were estimated to be 72% in low- and middle-income countries^9^. Moreover, 80% of mental disorders are estimated in low- and middle-income countries^9^. By 2030, it is projected that cancer deaths will increase by 45% from 7.9 million recorded in 2010 to 11.5 million^9^. Liver, stomach and cervical cancer are the most common cancers in developing countries^9^,^10^. Certainly, the increase in cancer prevalence in low-and middle-income countries eventually will lead to increase in mental disorders necessitating urgent attention.

The common risk factors for cancer development in developing countries are; an unhealthy lifestyle (including tobacco and alcohol use, poor diet, and physical inactivity), exposure to occupational (e.g. asbestos) or environmental carcinogens (e.g. indoor air pollution), exposure to radiation (e.g. ultraviolet and ionizing radiation), and infections (e.g. hepatitis B and human papilloma virus infection) 9. The World Health Organization (WHO) asserts that various cancers and mental health disorders are preventable with low-cost, high impact and evidence based interventions through primary prevention^9^. A study carried out to determine cancer survival rates in Uganda found that, the prognosis of cancer was lower for almost all forms of cancer compared to those in the developed world^11^. Cancer reports in Uganda show that over 60% of the cancer deaths occur in the first year after diagnosis and about 80% of the cancer patients die within two years of diagnosis^12^. The high cancer mortality rates in Uganda indicate that most of the cancer patients are diagnosed late when treatment is rarely effective^13^. Also, studies have demonstrated that individuals diagnosed with cancer suffer psychological distress such as depression (21%) and anxiety (29%) as co-morbid conditions^14^,^15^. Survivors of cancer diseases also suffer from psychological distress such as post-traumatic stress disorder (PTSD) (13%)^16^.

In resource-limited settings such as Uganda, family carers of cancer patients provide a crucial and an extensive care role and support in addition to that provided by health care providers. Most family carers of cancer patients report emotional challenges caused by just thinking about the chronic nature of the cancer, its treatment and prognosis^17^. Although the majority of the studies reviewed are not from Uganda, there is documented evidence of the caregiver role being stressful^19^. Stressful roles related to caregiving include: prolonged self-care tasks, administering prescribed treatment, dealing with patient symptoms and suffering, and coping with frequent hospital visits^18^,^19^. Caregiving roles may also lead to social, economic, psychological and physical challenges, including disruption of one's schedule and lifestyle, affects employment, change family roles and social life, as well as perceived as burdening and costly^19^,^20^. Carers of cancer patients may also encounter greater out-of-pocket expenses during this period, while others become less productive with less income^19^,^20^. Therefore, specialist psychological support services and interventions are imperative to address these challenges^9^.

Additionally, primary caregivers of cancer patients report depression and anxiety related to start of the palliative care phase to the terminal phase^4^,^21^. However, little is known about the prevalence of anxiety and depression among cancer carers at the cancer care and treatment facility in Uganda. Moreover, psychological services are not available for family carers to access at the Uganda Cancer Institute. This study was aimed to determine the prevalence of anxiety and depression among family carers of cancer patients and the related factors associated with abnormal levels of anxiety and depression at the cancer care and treatment facility in Uganda.

## Methods

**Design.** The study utilized a cross-sectional study design using a convenience sample of family carers of cancer patients receiving treatment at Uganda Cancer Institute (UCI). The study was set to determine the prevalence of anxiety and depression and the related factors associated with abnormal levels of anxiety and depression among family carers of cancer patients.

### Setting and sample

Family carers identified by cancer patients (inpatients and outpatients) in the cancer care and treatment facility, who consented voluntarily and were aged 18–60 years were selected for the study. Of 124, 119 (Response Rate [RR] = 96%) of the participants agreed and completed their participation in the study. The study was carried out at the Uganda Cancer Institute (UCI), which is part of the Mulago hospital in Kampala. Mulago Hospital is the National teaching, research and referral hospital with 1500 bed capacity. The Uganda Cancer Institute in Mulago hospital is one of the two semi-autonomous Institutes second to Uganda Heart Institute (UHI) established in 1967 as a center for research on childhood cancer, especially Burkitt's lymphoma. Currently, the UCI has integrated services for treating numerous malignancies in adults and children. The UCI serves as the largest Uganda's referral center for cancer care and treatment.

### Measurements

Data was collected using the Hospital Anxiety and Depression Scale (HADS) standardized tool developed by Zigmond and Snaith^22^ in1983. Prior to data collection, the tool was pilot tested with a group of ten family carers of cancer patients on the surgical ward 2A of Mulago hospital. The analysis of the self-rating scale from the pilot study demonstrated that the tool was clear and could be used to study family carers. Family carers in the pilot study did not participate in the main study at the UCI.

**Study instrument and measurements:** The HADS standardized tool was used to collect data from family cancer carers in the study. The tool has been widely used mainly with cancer patients and rarely with carers. The HADS standardized tool is a valid tool with a reliability of (α=0.8525 for anxiety subscale and α= 0.7784 for depression subscale). The HADS standardized tool comprises of 14 statements or responses that are used to measure anxiety and depression in both hospital and community settings^22^. The 4-point Likert scale standardized tool has seven (7) statements in the anxiety subscale and the other seven (7) in the depression subscale. Responses are categorized into level of depression (D) with scores of 0, 1, 2, 3 in ascending order where 0= severe depression and 3= no depression. Level of anxiety (A) has scores ranging from 3, 2, 1, 0 in descending order in which 3= severe form of anxiety and 0= no anxiety. Identification of anxiety and depression in an individual is calculated by adding up all the scores of seven items for anxiety and depression subscales respectively. A total score of 11–21 on the anxiety subscale indicates that one has abnormal level of anxiety, while the same total score of 11–21 on the depression subscale will indicate that one has an abnormal level of depression. Zero to seven total scores indicates normal levels of anxiety and depression, while 8–10 total scores indicate that someone has borderline abnormal levels of anxiety and depression. The tool was supplemented with self- administered demographic characteristic questionnaire to help in describing the characteristics of the sample.

### Data analysis

The Statistical Package for Social Sciences (SPSS) version 20 for Windows (SPSS, Chicago, USA) was used for data coding and processing. Simple descriptive statistics were run on all demographic variables to identify missing data and to determine the prevalence of anxiety and depression. Odds ratio (OR) with 95% confidence interval (CI) was used to evaluate the factors associated with abnormal levels of anxiety and depression. Both unadjusted and adjusted ORs were calculated and analyzed. All data were checked for completeness before data analysis and reporting.

### Ethical consideration

Prior to giving their consent, participating family carers were provided with information about the study and assured of anonymity and confidentiality of their information. The study protocol received ethical approval (REC REF: 2011-025) by the Ethics Review Board of Makerere University, Institutional Review Board of Mulago Hospital, and the Uganda Cancer Institute.

## Results

### Characteristics of family carers

In this study, all one hundred nineteen (119) family carers of cancer patients were eligible and completed their participation in the study. Sixty seven percent (67%) were female family carers. The mean age of the participants was 33 years (S.D. 10.69). Of the study participants, 32% of the family carers had attained primary level of education, 45% were married and 61% were employed. Forty one percent (41%) of the participants reported to be first degree relatives of the cancer patients other than parents. Sixty seven percent (67%) of the participants reported that care giving responsibility was burdening to them (Table 1).

**Table 1:** Socio-demographic characteristics and anxiety and depression status

Characteristic	All Respondents	With ALA	With no ALA	With ALD	With No ALD
**Age:** mean (standard deviation)	33.03 (10.69)	33.43 (10.52)	32.70 (10.90)	34.10 (12.12)	32.68 (10.28)
**Sex:** n (%)					
Male	39 (32.8)	18 (46.2)	21 (53.9)	12 (30.8)	27 (69.2)
Female	80 (67.2)	35 (43.8)	45 (56.3)	17 (21.3)	63 (78.8)
**Level of formal education:** n (%)	11 (9.2)				
None	38 (31.9)	6 (54.5)	5 (45.5)	2 (18.2)	9 (81.8)
Primary	34 (28.6)	18 (47.4)	20 (52.6)	12 (31.6)	26 (64.4)
Secondary	36 (30.3)	12 (35.3)	22 (64.7)	7 (20.6)	27 (79.4)
Tertiary		17 (47.2)	19 (52.8)	8 (22.2 )	28 (77.8)
**Marital status:** n (%)					
Single	55 (46.2)	20 (41.7)	28 (58.3)	12 (25.0)	36 (75.0)
Married	53 (44.5)	23 (43.4 )	30 (56.6)	15 (28.3)	38 (71.7)
Separated	11 (9.2)	10 (55.6)	8 (44.4)	2 (11.1)	16 (88.9)
**Employment:** n (%)					
Employed	73 (61.3)	29 (39.7)	44 (60.3)	18 (24.7)	55 (75.3)
Unemployed	28 (23.5)	15 (53.6)	13 (46.4)	7 (25.0)	21 (75.0)
Student	18 (15.1)	9 (50.0)	9 (50.0)	4 (22.2)	14 (77.8)
**Relationship to patient:** n (%)					
Parent	32 (26.9)	21 (65.6)	11 (34.4)	13 (40.6)	19 (59.4)
Other 1^st^ degree relatives	49 (41.2)	19 (38.8)	30 (61.2)	10 (20.4)	39 (79.6)
Extended family	32 (26.9)	11 (34.4)	21 (65.6)	4 (12.5)	28 (87.5)
Other	6 ( 5.0)	2 (33.3)	4 (66.7)	2 (33.3)	4 (66.7)
**Perception of the responsibility:** n (%)					
Burdening	80 (67.2)	42 (52.5)	38 (47.5)	23 (28.8)	57 (71.3)
Not burdening	39 (32.8)	11 (28.2)	28 (71.8)	6 (15.4)	33 (84.6)
**Burdened, accessing support:** n (%)					
Yes	46 (57.5)	21 (45.7)	25 (53.4)	12 (26.1)	34 (73.9)
No	34 (42.5)	21 (61.8)	13 (38.3)	11 (35.4)	23 (67.7)
(Not Burdened)	39 (32.8)	11 (28.2)	28 (71.8)	6 (15.4)	33 (84.6)
**Months of care giving:**					
mean (standard deviation)		11.28(16.5)	6.43(11.8)	7.42 (9.3)	8.97(15.5)

### Prevalence of abnormal level of anxiety among the participants

Forty five (45%) of the study participants had abnormal levels of anxiety (ALA) with total scores ranging from 11–20 on the anxiety sub-scale of the HADS standardized tool (Table 1). The male carers were most affected and expressed high levels of anxiety than their female counterparts (46.2% v.43.8%) (Table 1).

### Prevalence of abnormal levels of depression among the participants

Twenty six (26%) of the participants had abnormal levels of depression (ALD) with total scores ranging from 11–20 on the depression subscale of the HADS standardized tool (Table 1). Similarly, the male carers were most affected and reported high levels of depression than the female carers (30.8% v. 21.3%) (Table 1).

### Predictors and correlates of abnormal levels of anxiety

Multiple logistic regression analysis showed that, the type of relationship to the patient was significantly associated with abnormal levels of anxiety [ALA] (Table 2). Family carers who were “other first degree relatives” were 67% less likely to experience abnormal levels of anxiety (OR 0.33, p=0.02), compared to 73%- who are carers of extended family members (OR 0.27, p=0.01) (Table 2). There was no association between the abnormal levels of anxiety and other types of relationships (OR 0.26, p=0.16).

**Table 2:** Predictors and correlated of social demographic characteristics and anxiety

Character	Univariate			Multivariate		
	Odds Ratio	Confidence Interval	P value	Odds Ratio	Confidence Interval	P value
**Log age:**	1.33	0.42–4.21	0.62			
**Sex:** Male Female						
**Level of formal education:** None (ref) Primary Secondary Tertially	__ 0.75 0.45 0.75	__ 0.20–2.88 0.11–1.80 0.19–2.89	__ 0.68 0.26 0.67			
**Marital status:** Single (ref) Married Separated	__ 1.07 1.75	__ 0.49–2.37 0.59–5.21	__ 0.86 0.32			
**Employment:** Employed (ref) Unemployed Student	__ 1.75 1.52	__ 0.73–4.21 0.54–4.28	__ 0.21 0.43			
**Months of providing care:**						
**Relation to patient:** Parent (ref) Other 1^st^ degree relatives Extended family Other	__ 0.33 0.27 0.26	__ 0.13–0.84 0.10–0.77 0.04–1.66	__ 0.02* 0.01* 0.16	__ 0.34 0.29 0.31	__ 0.13–0.88 0.10–0.84 0.16–0.88	__ 0.26 0.02* 0.23
**Perception of the responsibility:** Burdening (ref) Not burdening	__ 0.36	__ 0.16–0.81	__ 0.01*	__ 0.38	__ 0.16–0.88	__ 0.02*
**Burdened, accessing support:** Yes No (Not Burdened)						

Significant at p≤ 0.05*, p≤0.01**, p≤ 0.001*** CI 95%

Adjusting for perception of responsibility and type of relation to the patient, were significantly associated with abnormal levels of anxiety. Carers who were relatives in the extended family settings were 71% (OR 0.29, p=0.02) less likely to experience abnormal levels of anxiety (Table 2). There was no significant association between “other first degree relatives” (OR 0.34, p=0.26) and “other types of relationships” (OR 0.31, p=0.23) (Table 2). Results showed that the type of relation to the patient was significantly associated with abnormal levels of anxiety (OR 0.38, p=0.02) (Table 2).

### Predictors and correlates of abnormal level of depression

Results showed that, carer relationship to the patient was significantly associated with abnormal levels of depression. Carers who were other first degree relatives were 63% (OR 0.37, p=0.05) less likely to experience abnormal levels of depression. Similarly, carers of relatives in extended family settings were 79% (OR 0.21, p=0.02) less likely to experience abnormal levels of depression. There was no significant difference in the abnormal levels of depression for other types of relationships (OR 0.73, p=0.74). Other demographic factors (age, sex, level of formal education, marital status, employment status and period of providing care) did not demonstrate any significant association with abnormal levels of depression. The perception of care responsibility burden to the carers showed no significant association with ALD (Table 3). Furthermore, there was a significant association between the caregiver relationship to the patient and abnormal levels of depression. Carers in the extended family showed abnormal levels of depression (OR 0.22, p= 0.02). There was no significant association of abnormal levels of depression and perception of caregiving responsibility.

**Table 3:** Predictors and correlated of social demographic characteristics and depression

Character	Univariate			Multivariate		

	Odds	Confidence	P value	Odds	Confidence	P
	Ratio	Interval		Ratio	Interval	value
**Sex:**						
Male						
Female						
**Level of formal education:**						
None	---	---	---			
Primary	2.08	0.39–11.12	0.39			
Secondary	1.17	0.20–6.67	0.86			
Tertially	1.29	0.23–7.19	0.78			
**Marital status:**						
Single	---	---	---			
Married	1.18	0.49–2.87	0.71			
Separated	0.38	0.08–1.87	0.23			
**Employment:**						
Employed	---	---	---			
Unemployed	1.02	0.37–2.79	0.97			
Student	0.87	0.25–2.99	0.83			
**Relation to patient:**						
Parent	---	---	---	---	---	---
Other 1^st^ degree relatives	0.37	0.14–1.01	0.05*	0.39	0.14–1.05	0.06
Extended family	0.21	0.59–0.74	0.02*	0.22	0.06–0.79	0.02*
Other	0.73	0.12–4.59	0.74	0.87	0.13–5.71	0.89
**Perception of the** **responsibility:**	---	---	---	---	---	---
Burdening	0.45	0.17–1.22	0.12	0.38	0.14–1.05	0.15
Not burdening						

Significant at p≤ 0.05*, p≤0.01**, p≤ 0.001*** CI 95%

### Perception of the caregiver responsibility

The study demonstrated that the perception of care responsibility was significantly associated with abnormal levels of anxiety. Family carers of cancer patients were 64% less likely to experience care-related burden responsibility (OR 0.36, p=0.01) (Table 2). Other demographic characteristics such as age, sex, level of formal education, employment, marital status and period of care did not demonstrate any significant association with abnormal levels of anxiety

## Discussion

This study was restricted to family carers of cancer patients. In Uganda's population census, the proportion of females is greater than that of males therefore; our study findings are not surprising. Still in Uganda, the majority of the families live in extended family structures. Therefore, it is not unusual to find female carers in the caregiving role. Moreover, the majority of the societies recognizes and trusts the female carers to be more caring than their male counterparts^23^. It is believed that thecaregiving role requires patience, commitment and is time consuming^23^. Families with chronic sick persons such as those with cancer have to decide on somebody who will take full responsibility of the sick considering the burden of caregiving roles^17^. When assigning the caring roles, the nature of employment of the cancer carer for optimal care is an important aspect to consider^19^. Moreover, dividing household management roles and caregiving tasks to other relatives is equally important to relieve some stress from primary caregiver^19^. In this study, majority of the carers were employed and this may explain why carers expressed high levels of anxiety and depression coupledwith other pressing needs^19^. The biggest challenge of caring for cancer patient relates to knowing that cancer disease has a poor prognosis, thus a cause of anxiety and depression among cancer carers than non-cancer caregivers^19^. Furthermore, the caregiving role presents with other challenges such as unexpected costs spent on drugs, feeding and other related expenses at the hospital^19^.

Culturally, in Uganda, single marital status does make one vulnerable for being selected as a carer of the cancer patient in the family^24^. The relationship with the patient may also be used to determine who is suitable for the caregiving role. Closely related relatives are prioritized over other distant relatives since cancer patients tend to report discomfort in the care of non-relative caregivers^24^. It is therefore not surprising that the majority of carers were first degree relatives of the patient in the current study^24^. The findings of this study are consistent with findings of other studies that reported that strong family ties to be imperative in offering support during distress^25^. It is contended that family members have a filial obligation to take care of the patient^23^,^24^.

Approximately 85% of Uganda's population predominately live in rural setting^26^. The majority of the cancer patients and their carers in the cancer care and treatment at Uganda Cancer Institute are mainly referrals. These rural based carers are usually burdened by distance, medication costs and therefore, are likely to report high levels of anxiety and depression^19^. The findings are consistent with a study by Grov et al^21^ that found that 45% of caregivers of cancer patients had HADS-defined anxiety responses. Contrarily, our findings are greater than those found by Grunfeld and colleagues^4^, who found a prevalence of 35% using the HADs standardized tool. The surprising findings could be explained by the smaller number of male carers in our study compared to their female carer counterparts. Numerous studies have on the other hand found that the male carers are more likely to suffer from anxiety and depression than their female counterparts^25^. In particular, anxiety and depression disorders could be identified when there is an advanced illness requiring palliative treatment and without a support network^25^.

Similarly, depression was prevalent among family carers of cancer patients at UCI. The results from our study are seemingly lower (26%) compared to 30% found in a study by Grunfeld et al^4^. The lower prevalence rates in Uganda could be explained in part by the family social structures characterized by living in extended families. Additionally, family carers and their patients receive spiritual support from church members and other individuals who visit the hospital. This gives a lot of hope to the sick and their carers^27^. Although some studies have reported lower prevalence rates of depression of up to 18% among cancer caregivers^21^, contrarily, our study found higher prevalence of depression. This could be explained in part by the fact that most cancer patients in Uganda come to seek health care in late stages of cancer causing more distress to their carers^19^. The findings of our studies are supported by reports from other studies that documented that primary caregivers of patients with terminal phase of cancers have increased scores of depression levels^4^,^21^.

Although studies have shown that caregiving of cancer patients may be rewarding^19^, our study found that being a first degree relative or family carer was significantly associated with abnormal levels of anxiety and depression. This means that carers needed a strong social support network to overcome their burden^19^,^24^. The current study did not show other demographic characteristics such as age, level of education, employment to be associated with abnormal levels of anxiety and depression. This could be explained by the fact that many societies in Uganda perceive caregiving role to be rewarding^24^. Family carers in the current study reported that they were burdened by the caregiving responsibility thus the observed abnormal levels of anxiety. The findings are consistent with findings from other studies that showed that the caring responsibility affected the social life of caregivers through disrupting of their schedules and lifestyles^17^,^20^.

## Study limitations

Firstly, it was conducted in one center in an urban setting. This could have influenced the self-reported responses about anxiety and depression levels from the carers. The use of a convenient sample of family carers could affect the generalizability of the results. We used only one standardized tool (HADS) to assess the abnormal levels of anxiety and depression without assessing clinical presentations which may cause over rating or incorrect screening methods of the anxiety and depression among this group.

Implication for practice and policy: Health care providers in primary and tertiary facilities need to recognize and screen for anxiety and depression among family carers of cancer patients. Integrating psychosocial services specifically for family carers is crucial for improved quality of life for both patients and carers.

## Conclusion

There is a high prevalence of anxiety and depression among family carers of cancer patients receiving cancer treatment at Uganda Cancer Institute. Both anxiety and depression were found to be prevalent mostly in male carers than their female counterparts. Being a first degree relative family carer and a carer in an extended family was associated with abnormal levels of anxiety and depression. Caregivers' perception of caregiving responsibilities was significantly associated with abnormal levels of anxiety. Health care providers in both primary and tertiary facilities need to recognize and screen for anxiety and depression among family carers of patients with cancer, so as to provide appropriate psychological therapies that promote quality of life of the carers and patients. Incorporating evidence based psychological therapies targeting family carers into usual care of cancer patients is imperative.

## Abbreviations

ALA: Abnormal Level of Anxiety
ALD: Abnormal Level of Depression
HADS: Hospital Anxiety and Depression Scale (HADS)
RR: Response Rate
UCI: Uganda Cancer Institute
